# Public School Dental Clinics a Possibility in Each Community; a Practical Plan of Securing and Maintaining Them

**Published:** 1914-04-15

**Authors:** W. G. Ebersole


### PUBLIC SCHOOL DENTAL CLINICS A POSSIBILITY IN EACH COMMUNITY; A PRACTICAL PLAN OF SECURING AND MAINTAINING THEM.

BY W. G. EBERSOLE, M.D., D.D.S.

Delivered before the Fourth International Congress on School Hygiene, Buffalo, N. Y., August 27, 1913.

One of the most important divisions of the work of the National Mouth Hygiene Association is that which deals with the establishment of dental clinics, hospitals or dentariums, where proper care and treatment may be afforded those who are unable to care for themselves; providing not only dental service but furnishing tooth toilet articles which will enable the poor to take care of the "gateway" to the human system in a manner which will permit of the highest development from a physical standpoint.

It is the purpose of the National Mouth Hygiene Association to not only establish dental clinics for the care and treatment of the worthy poor, but to raise an endowment fund sufficient to guarantee the successful operation of these clinics indefinitely.

Our campaign embraces all that has meant success in other money raising campaigns and in addition it embraces a feature which if employed alone would result in the successful raising of funds to meet the work we are undertaking.

Our campaigns are waged in the interest of the school child and therefore the school child is the hub of the campaign.

The National Mouth Hygiene Association stands for the removal of the shackles that are binding the school children down to sin, sickness and death by forcing them into schools where they are surrounded by conditions which, if we accept the statement of the Michigan State Board of Health, is destroying 27.6 per cent. of the public school teachers, who preside over these children, from tuberculosis, which is only one of the many diseases that are destroying thousands of lives annually.

The Association is not interested alone in the school child but in the teaching profession as well, and it does not stop there. It is interested in the health, strength, and working efficiency of every individual in the United States.

But for the present we must deal with the school child and those interested in its welfare.

The work of the Association is educational, and it employs the four greatest educational institutions—The Public School, The Public Press, The Public Platform, and the Motion Picture. The greatest of these is the Public School, and it is through the school child that we hope to accomplish most.

It is for this purpose that the Association has undertaken to establish dental clinics in the schools of the country.

The National body is not interested in the dental clinic except as an educational feature. The local auxiliary on the other hand is interested in both the educational feature and in the benefit that it will do the community.

The clinic cares for the indigent, dealing with the few. The National Mouth Hygiene movement must deal with all the people, the rich, the poor, and those of the middle class. All walks of life need Mouth Hygiene instruction.

Therefore in the employment of the campaign for raising funds, the plan embraces a method and system which will bring to every individual in the community an invitation to become a part of and contribute to this great movement in the interest of the health, strength, and working efficiency of humanity.

The first move necessary to secure the co-operation of the National Mouth Hygiene Association in your local work is the formation of a local auxiliary to that body in your community.

In accomplishing this end the interested party will find in the early stages that the people most willing to co-operate will be the dentists, physicians and school teachers, and it is from these professions that the foundations must be secured for a successful local auxiliary.

Following the organization of the local auxiliary an application must be filled with the Secretary-Treasurer of the National body, requesting the installation of a publicity campaign in the community in which such auxiliary was formed. Applications will be filed and acted upon in the order received.

When the Association is ready to operate in any given community the preliminary publicity work will be directed by one of the officers of the National Mouth Hygiene Association, who will visit the city where the campaign is to be conducted in advance of the field Secretary, and it will be his purpose to arouse public interest through the newspapers, from the public platform, and in other educational ways.

That some definite idea may be had as to the manner in which the public interest may be aroused will say that these officials will cause bacterial tests to be made in public places, such as school buildings, street cars, theaters, etc.

He will also make tests in the mouths of the school children, sterilizing mouths and then employing gauze masks, transferring the children and then exposing them in the various public places for the purpose of showing how quickly the sterile mouth may become infected and the various kinds of bacteria that will be found in these mouths after a few minutes exposure in public places.

Following this will be a period in which the Association employs newspaper advertising.

At this time the Association will send into the field a professional organizer, whose business it will be to raise the money to secure a clinic and endow it for an indefinite period of time.

The preliminary work that this organizer must do is to secure the support and co-operation of the health and educational authorities in the community. The co-operation and support of all municipal and civic organizations must be secured.

Following this the teaching profession of the community must be organized and their support and co-operation obtained.

When this has been done we are ready to start with the financial campaign. This will cover a period of a week of active work.

It is utterly impossible for me to go into full details relative to the organizing and conducting of such a campaign. Let me say, however, that in addition to the solicitation of large contributions the local auxiliary, which has the work in charge, will endeavor to raise a large percentage of this money by waging the strongest kind of a campaign for membership; the dues to be turned into the general fund.

The membership in the local auxiliary is composed of two classes—"contributing members" and "working members." The first class is composed of those members who make contributions of $1.00 or more. The second class is composed of those who are not able to make financial contribution but are willing to devote a definite amount of time to the work of the Association in carrying out its plans and policies in the community.

To the latter class belongs the school child in whose interest the campaign is conducted.

Working in the interest of the school child of the community the plan embraces, in so far as practical, the employment of the school children as workers in the field.

The younger children will be used in the distribution of a booklet and other educational matter, making a strong appeal for support of the movement.

The booklet used will be entitled the "Cry of the Shackled Child, A Plea to Aid Mentally Crippled School Children, whose proper development is held back by bad teeth and unhealthy mouths".

This booklet will contain a strong appeal that cannot fail to reach the heart and create a deep interest in the success of the work undertaken.

For the purpose of giving you some idea of the contents of a booklet of this kind I beg to quote the following extract, which is taken from the inside of the front cover:—

> "THIS booklet has been placed in your hands by a school child. In justice to your juvenile friends and relatives, the least you can do is to read it.
>
> "Educators, clergymen, civic workers, city officials and all other public-spirited citizens have approved the movement it promotes.
>
> "At any rate, it will present some facts to you that will surprise you and take only a few minutes of your time in reading it.
>
> "In a few days you will be called upon to aid this great work by becoming a member of the National Mouth Hygiene Association's local auxiliary. You can help materially by giving the young solicitor courteous attention. If you cannot see your way clear to join the children, at least do not dishearten the child who calls upon you by an abrupt or harsh rebuff. He is working for humanity and certainly deserves encouragement."

This booklet explains fully the purpose of the campaign and the need of co-operation.

A day or two following the distribution of these booklets an older child calls soliciting membership in the local auxiliary, collecting the fee of $1.00, and issuing a membership card which in addition to calling for educational literature contains a coupon which, when presented at any drug store in the city, entitles the bearer to a full "Dollar's worth of tooth toilet articles."

The booklet which has been distributed explains to the individual, whom the child solicits, that the National Mouth Hygiene Association has placed in operation a plan which makes it possible for its local auxiliaries, that are conducting campaigns, to buy and place in the hands of their members packages of the highest grade of tooth toilet articles, which ordinarily retail for $1.00, and by the transaction be able to set aside 50 per cent. of the membership fees for the endowment and maintenance of dental clinics.

These membership packages contain four large tubes or boxes of tooth paste or powder, such as ordinarily retail at 25 cents per tube or box.

These "four tube" or "four box" packages are supplied to local auxiliaries in a manner which enables them to place same in the hands of the druggist at a cost of about 40 cents per package. The druggist is allowed 10 cents per package for reclaiming the membership coupons. This makes the $1.00 membership package," delivered to the member, cost the local auxiliary 50 cents.

The new member for the $1.00 paid, receives a full membership in the local auxiliary and a "$1.00's worth of tooth toilet articles;" and by the transaction places in the hands of the local auxiliary the profit which otherwise goes to the manufacturer of tooth toilet articles. In this case the profit amounts to 50 cents on the $1.00 and is retained in the community and devoted to endowing and maintaining school clinics.

The National Association has also arranged a plan whereby the same tooth toilet articles may be supplied through the regular commercial channels on a basis which will enable the community to reap the benefit of the profit from such sales.

This is done by the placing in the carton of each 25 cent package a metal disc, which when collected by the local auxiliary, entitles that organization to collect 5 cents for each disc to be used in its local work, or in maintaining dental clinics.

The difference between the profit accruing to the auxiliary from the "$1.00 membership package" and the four 25 cent packages sold through regular trade represents the difference between supplying large quantities to the local auxiliaries at cost and the commercial and overhead charges in handling these goods through the regular commercial channels.

This is a co-operative method which permits the people in a community to turn the profit that now goes to the tooth toilet manufacturers of the country into a local fund for the purpose of not only establishing school dental clinics and other clinics, but supplying tooth toilet articles to the poor of the community. Such a plan would enable these individuals to have at their command methods and means to care for themselves in a manner which would permit them to reap the benefits of healthy mouth conditions and thus remove the possibility of their becoming a public menace through neglect or inability to care for the mouth properly.

In order to give some idea of the tremendous profit that accrues from the manufacture and sale of these articles, I wish to say that some of the leading manufacturers have been and are paying as much as from five to seven thousand dollars an issue for the back cover page of some of the most popular magazines.

I can readily understand that immediately within the mind of the skeptic or doubter there arises the question of graft or the attempt to advertise some tooth toilet article; but I wish to announce to the world at large that the plan has been drawn and safeguarded in such a manner as to make it absolutely impossible for any one associated with the National Mouth Hygiene Association or any of its auxiliaries to receive one penny of profit or graft from the transaction.

I wish also to state in the most emphatic terms that no manufacturer of tooth toilet articles is directly or indirectly interested in the promotion of this plan.

There are four things which have led to the adoption of the plan herein suggested:—

First: The need of funds to carry on the work of the Association in the various communities.

Second: The success attained in raising funds for Y. M. C. A. buildings, hospitals, dispensaries, etc.

Third: The popularity and success attained in raising funds through the sale of Red Cross Stamps.

Fourth: The fact that the National Mouth Hygiene Association's campaign is unique in that every effort put forth to educate the public as to the importance of Mouth Hygiene creates a demand for tooth toilet articles.

In adopting the plan for supplying tooth toilet articles as herein outlined, the Association has combined the raising of funds and the supplying of a need; and it is believed that this feature will become as popular and more effective than the Red Cross Stamp feature of the Anti-Tuberculosis League.

In conclusion let me say that the whole success of the plans and policies mentioned herein depends both upon the employment of thoroughly competent experts in the different lines of work and upon the presentation of the matter in a way which will convince the public that this is absolutely a philanthropic and economic proposition.

The National Mouth Hygiene Association has undertaken to do this work in the interest of humanity and it guarantees that every step taken and every act committed will bear the most rigid inspection and investigation; for upon the honesty of purpose and purity of action must rest not only the success of the scheme but the good name of the Association and those associated with it.

In presenting this paper for the consideration of the general public I wish to acknowledge the aid and support given by the board of Governors of the National Mouth Hygiene Association; and particularly that of Dr. T. W. McFadden of Wilkinsburg, Pa., who has been conducting a series of experiments as to the efficiency of the proposed plan for raising funds from the sale and distribution of tooth toilet articles. The work done by Dr. T. W. McFadden and the results obtained have been simply marvelous.

